# A physiologic overview of the organ-specific transcriptome of the cattle tick *Rhipicephalus microplus*

**DOI:** 10.1038/s41598-020-75341-w

**Published:** 2020-10-26

**Authors:** Lucas Tirloni, Gloria Braz, Rodrigo Dutra Nunes, Ana Caroline Paiva Gandara, Larissa Rezende Vieira, Teresa Cristina Assumpcao, Gabriela Alves Sabadin, Renato Martins da Silva, Melina Garcia Guizzo, Josias Alves Machado, Evenilton Pessoa Costa, Daniele Santos, Helga Fernandes Gomes, Jorge Moraes, Maria Beatriz dos Santos Mota, Rafael Dias Mesquita, Milane de Souza Leite, Patricia Hessab Alvarenga, Flavio Alves Lara, Adriana Seixas, Rodrigo Nunes da Fonseca, Andrea C. Fogaça, Carlos Logullo, Aparecida Sadae Tanaka, Sirlei Daffre, Pedro L. Oliveira, Itabajara da Silva Vaz, José M. C. Ribeiro

**Affiliations:** 1 Centro de Biotecnologia, Universidade Federal do Rio Grande do Sul, Porto Alegre, Brazil; 2 Tick-Pathogen Transmission Unit, Laboratory of Bacteriology, National Institute of Allergy and Infectious Diseases, Hamilton, USA; 3 Departamento de Bioquímica, Instituto de Química, Universidade Federal do Rio de Janeiro, Rio de Janeiro, Brazil; 4 Laboratório de Bioquímica de Artrópodes Hematófagos, Instituto de Bioquímica Médica, Universidade Federal do Rio de Janeiro, Rio de Janeiro, Brazil; 5 Instituto de Biodiversidade e Sustentabilidade NUPEM, Universidade Federal do Rio de Janeiro, Rio de Janeiro, RJ Brazil; 6 Laboratorio de Bioquímica de Resposta ao Estresse, Instituto de Bioquímica Médica, Universidade Federal do Rio de Janeiro, Rio de Janeiro, Brazil; 7 Laboratório de Microbiologia Celular, Instituto Oswaldo Cruz, Fundação Oswaldo Cruz, Rio de Janeiro, Brazil; 8 Departamento de Bioquímica, Escola Paulista de Medicina, Universidade Federal de São Paulo, São Paulo, SP Brazil; 9 Departamento de Farmacociências, Universidade Federal de Ciências da Saúde de Porto Alegre, Porto Alegre, RS Brazil; 10 Departamento de Parasitologia, Instituto de Ciências Biomédicas, Universidade de São Paulo, São Paulo, Brazil; 11 Instituto Nacional de Ciência e Tecnologia-Entomologia Molecular, Rio de Janeiro, RJ Brazil; 12 Vector Biology Section, Laboratory of Malaria and Vector Research, National Institute of Allergy and Infectious Diseases, Rockville, USA

**Keywords:** Biochemistry, Cell biology, Computational biology and bioinformatics, Molecular biology

## Abstract

To further obtain insights into the *Rhipicephalus microplus* transcriptome, we used RNA-seq to carry out a study of expression in (i) embryos; (ii) ovaries from partially and fully engorged females; (iii) salivary glands from partially engorged females; (iv) fat body from partially and fully engorged females; and (v) digestive cells from partially, and (vi) fully engorged females. We obtained > 500 million Illumina reads which were assembled de novo, producing > 190,000 contigs, identifying 18,857 coding sequences (CDS). Reads from each library were mapped back into the assembled transcriptome giving a view of gene expression in different tissues. Transcriptomic expression and pathway analysis showed that several genes related in blood digestion and host-parasite interaction were overexpressed in digestive cells compared with other tissues. Furthermore, essential genes for the cell development and embryogenesis were overexpressed in ovaries. Taken altogether, these data offer novel insights into the physiology of production and role of saliva, blood digestion, energy metabolism, and development with submission of 10,932 novel tissue/cell specific CDS to the NCBI database for this important tick species.

## Introduction

The cattle tick *Rhipicephalus microplus* is a one-host tick, which preferentially feeds on bovines, and it is considered the most harmful cattle parasite^1,2^. The economic losses associated with *R. microplus* parasitism are due to direct effects of the tick itself, which causes skin injuries and long-standing blood loss, leading to anemia and reduction of both weight gain and milk production, or are produced indirectly via transmission of tick-borne pathogens such as *Babesia* spp. and *Anaplasma marginale*^3^. In spite of its huge impact on the economy, current tick control strategies still rely mostly on the use of chemical acaricides, even though selection of resistant tick populations to major used acaricides has been confirmed^4,5^. This is recognized as a worldwide drawback to successful tick control. Immunization of cattle against *R. microplus* and other ticks has been recognized as an alternative approach against chemical control strategy^6^. Thus, a deeper understanding of tick physiology is needed as a means to find molecular targets that can be useful in the development of novel tick control methods.

Transcriptomic analyses have contributed with valuable information regarding the physiology of several parasites, including ticks^7,8^. The genomes of *Ixodes persulcatus*, *Ixodes scapularis*, *Haemaphysalis longicornis*, *Dermacentor silvarum*, *Hyalomma asiaticum*, *Rhipicephalus sanguineus*, and *Rhipicephalus microplus* have been sequenced and now are helpful resources for studying tick physiology and biology^9–11^. The genome of the cattle tick *R. microplus* was estimated to be 7.1 Gbp in length and consists of approximately 70% repetitive DNA^12^. Recently, a cattle tick genome draft was reported^13^ using a hybrid sequencing and assembly approach to capture the repetitive fractions of the genome. According to this study, the *R. microplus* genome assembly is composed of 51.4% repetitive sequences, containing 38,827 putative *R. microplus* gene loci, of which 24,758 are protein coding genes^13^. In spite of the large number of loci and of the significant advance provided by this genome assembly, based on the proportions of highly conserved single copy genes found in all arthropods, the degree of completeness of this assemblage was estimated to be about 41%, still leaving a vast open field for gene discovery in this organism. Recently, a remarkable publication reported the genome assembly of six tick species, including that of *R. microplus*^11^. A BUSCO analysis of the predicted proteome of this tick indicated a 51.8% of complete and single BUSCOs, an improvement over the assembly of Barrero et al.^13^.

Currently, there are several studies describing the genome^9–11,13^*,* transcriptomes^14–17^*,* and proteomes of ticks^18,19^*.* Based on these datasets, many different experiments have been carried out that elucidated tick-host^20^ or tick-pathogen interactions^18,19^, and can support the development of new control strategies^15^. Taken together, these reports highlighted the complexity of tick physiology mechanisms comprising both known and novel proteins participating in multiple cellular pathways. Recently, one study in *Ixodes ricinus* transcriptome shows that the genes involved in cuticle formation, chitin metabolism, and blood digestion enzymes are more related to fed stages. The basic energy metabolism pathway genes are more actively expressed during unfed stages, egg development, and embryogenesis^21^. In another recently published study, an integrated *R. microplus* transcriptome and proteome analyses of larvae and salivary glands of nymphs, males and females feeding on susceptible and resistant bovine hosts showed that the expression of genes involved in the host-parasite interaction are associated with host immune activation profile^22^.

Here, aiming to obtain a further insight into *R. microplus* transcriptome, we used RNAseq to carry out a study of tissue differential gene expression: (i) embryos from 1, 3, 5, 7, 9, 11, 13 day-old eggs; (ii) ovaries from partially and fully engorged females; (iii) salivary glands from partially engorged females; (iv) fat body from partially and fully engorged females; and (v and vi) digestive cells from partially and fully engorged females. Reads from each library were mapped back into assembled transcriptome, giving us a view of gene expression in different tissues. Taken altogether, this study offers additional transcriptomic data for this important tick species. The data illustrate the dynamic gene expression changes during the parasitic phase and the embryo development of *R. microplus* and reveals sets of genes that have a highly tissue-specific expression profile, pointing to pathways that may perform essential roles in the physiology of these tissues. These findings could be useful for future studies of tick physiology, contributing to the development of new tick control methods, as well as new biotechnological and pharmacological applications.

## Results and discussion

### An overview of *R. microplus* transcriptome

In this study, aiming to obtain an insight into the differential gene expression profiles associated with major aspects of the physiology of *Rhipicephalus microplus,* we used RNA-seq to carry out a transcriptome study:(i) embryo from 1, 3, 5, 7, 9, 11, 13-day-old eggs (EMB); (ii) ovaries (OV) from partially and fully engorged females; (iii) salivary glands (SG) from partially engorged females; (iv) fat bodies (FB) from partially and fully engorged females; and (v) digestive cells from partially engorged females, and (vi) fully engorged females (DIG-P and DIG-F, respectively). We obtained > 500 million raw reads, and after removal of Illumina primers and trimming of low quality bases, we obtained > 190 million high quality reads (Table 1). High-quality clean reads were assembled de novo using Abyss and Trinity assemblers. The resulting assemblies were then assembled together using a pipeline of iterative and parallelized BLASTn and CAP3; where BLASTn with decreasing word sizes (from 300 to 60) fed the CAP3 assembler through 15 iterations, producing 179,859 contigs longer than 150 bp and with more than 5 reads (Table 2). CDS were extracted based on similarities to known proteins on public databases or by presence of a signal peptide sequence^14^. Using this strategy, a total of 20,900 CDS larger than 150 nucleotides (nt) were obtained (Table 2), and 18,857 of which were annotated in different functional categories (Table 3 and Table S2). While some contigs were truncated and/or fragmented, 10,932 sequences were over 50% full length (based on their similarities to available Acari proteins) and were submitted to the NCBI (GHWJ01000000). A BUSCO analysis^23^ of the transcriptome indicated 54% complete and single copy BUSCOs, a better result than the 41% found for the assembly of Barrero et al.^13^ and 51.8% found for the assembly of Jia et al.^11^.

**Table 1:** Description of libraries used in the *Rhipicephalus microplus* transcriptome analysis.

SRA genbank number	Library	Tissue/stage	Reads (filtered)
SRR1187017	EMB	Embryos	15,050,820
SRR1187010	OV	Ovaries from partially and fully engorged adult females	34,468,825
SRR1187007	SG	Salivary glands from partially engorged adult females	33,105,548
SRR1187013	FB	Fat bodies from partially and fully engorged adult females	24,683,768
SRR1187012	SYN	Synganglion from partially and fully engorged adult females	26,309,385
SRR1187005	DIG-P	Digestive cells from partially engorged adult females	26,190,424
SRR1186998	DIG-F	Digestive cells from fully engorged females	30,562,811
	Total		190,371,581

**Table 2:** Statistics of *Rhipicephalus microplus* transcriptome assembly.

Statistic	Assembly
Number of assembled contigs	179,859
Number of CDS	20,900
Total size of CDS	28,459,316
Shortest CDS (nt)	150
Longest CDS (nt)	5772
L50 CDS size (nt)	483
150–200 (nt)	2766
201–300 (nt)	3848
301–400 (nt)	2353
401–500 (nt)	1724
501–1000 (nt)	5572
1001–2000 (nt)	3929
2001–3000 (nt)	592
> 3000 (nt)	116

**Table 3:** Functional classification and expression levels of the CDS extracted from the de novo assembly of *Rhipicephalus microplus* transcriptome.

Class	CDS	RPKM	CDS (%)	RPKM (%)
Unknown	4126	4,454,604	21.9	30.8
Secreted	3263	2,500,454	17.3	17.3
Metabolism, energy	263	1,364,735	1.4	9.4
Signal transduction	1225	667,446	6.5	4.6
Unknown, conserved	1435	628,726	7.6	4.3
Extracellular matrix/cell adhesion	308	501,770	1.6	3.5
Protein synthesis machinery	516	476,686	2.7	3.3
Transposable element	729	440,941	3.9	3.0
Transcription machinery	1208	393,739	6.4	2.7
Protein modification machinery	508	295,577	2.7	2.0
Metabolism, lipid	413	275,046	2.2	1.9
Secreted proteinase inhibitor	148	267,833	0.8	1.9
Protein export machinery	568	242,176	3.0	1.7
Immunity	210	212,390	1.1	1.5
Metabolism, carbohydrate	272	202,860	1.4	1.4
Nuclear regulation	391	196,891	2.1	1.4
Pathogen origin	14	191,693	0.1	1.3
Oxidant metabolism/detoxification	386	176,584	2.0	1.2
Cytoskeletal	383	158,558	2.0	1.1
Proteasome machinery	493	155,816	2.6	1.1
Transporters/storage	531	142,754	2.8	1.0
Secreted, lipocalins	252	128,862	1.3	0.9
Secreted protease	88	104,916	0.5	0.7
Signal transduction, apoptosis	83	56,990	0.4	0.4
Metabolism, amino acid	133	44,282	0.7	0.3
Secreted, mucins	26	36,072	0.1	0.2
Transcription factor	130	34,192	0.7	0.2
Metabolism, nucleotide	178	33,258	0.9	0.2
Vertebrate origin	262	24,904	1.4	0.2
Viral	124	21,701	0.7	0.1
Storage	34	16,725	0.2	0.1
Metabolism, intermediate	71	16,710	0.4	0.1
Bacterial	37	5217	0.2	0.0
Nuclear export	38	3488	0.2	0.0
Secreted, toxin	8	2051	0.0	0.0
Secreted, immunity	3	614	0.0	0.0
Total	18,857			

The 18,857 CDS identified in the current study were searched against the predicted translated sequences from the *R. microplus* genome^13^ (24,758 CDS) using BLASTP. Over 9198 (48%), 10,308 (54%), and 13,692 (72%) of protein-coding sequences in the transcriptome had at least one significant BLASTP hit when using E-values < 1e^–10^, < 1e^–5^, or < 1, respectively (Table S1).

**Figure 1:**
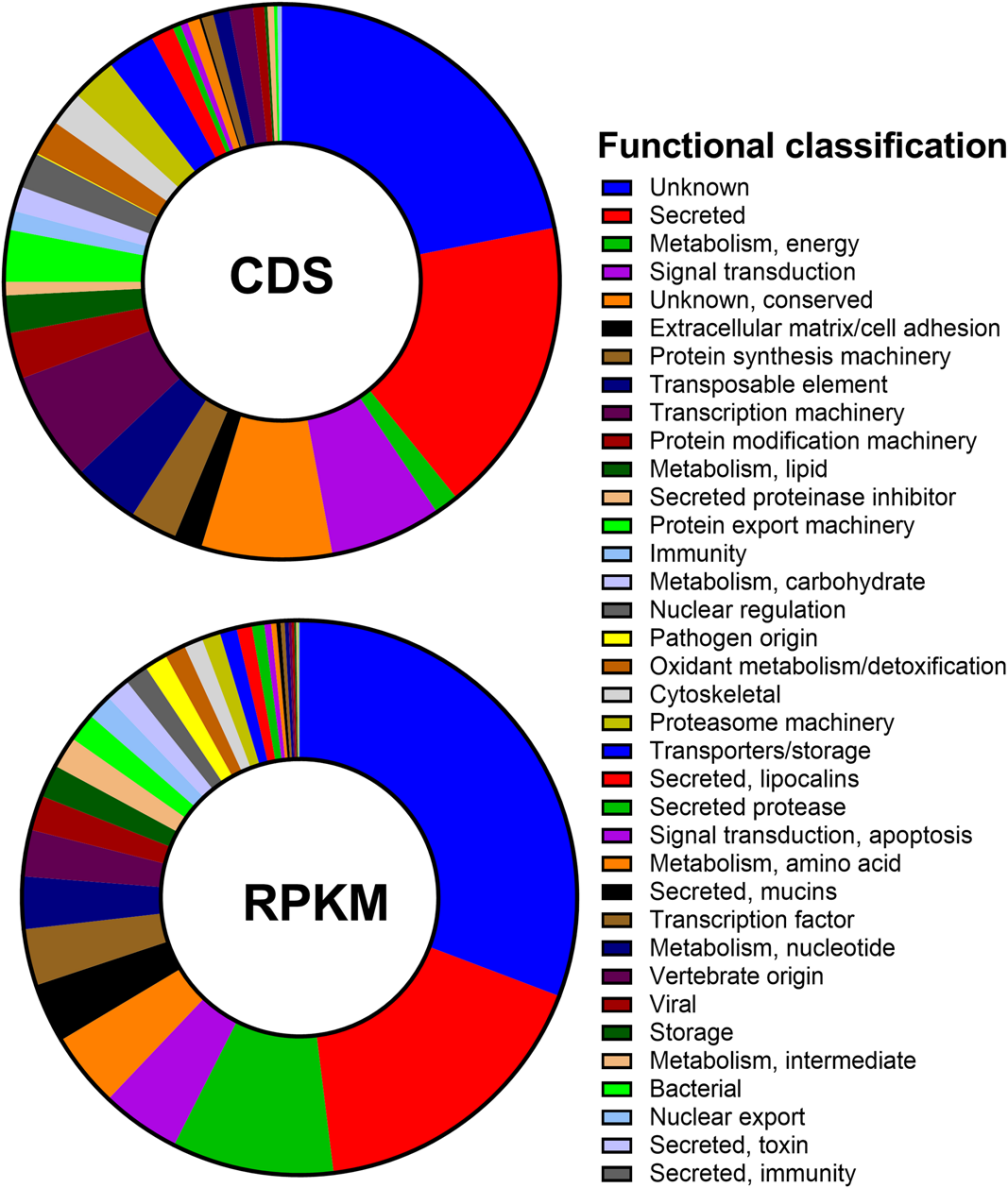
Functional classification of extracted coding sequences (CDS) from the *Rhipicephalus microplus* transcriptome. In CDS, the proportion of the total number of different CDS encoding a polypeptide of the same predicted function are compared to the total number of CDS found in our transcriptome. In RPKM, the proportion of the total RPKM number of different sequence reads assigned to CDS encoding a polypeptide of the same predicted function are compared to the total RPKM number of mapped sequences in our transcriptome.

**Figure 2:**
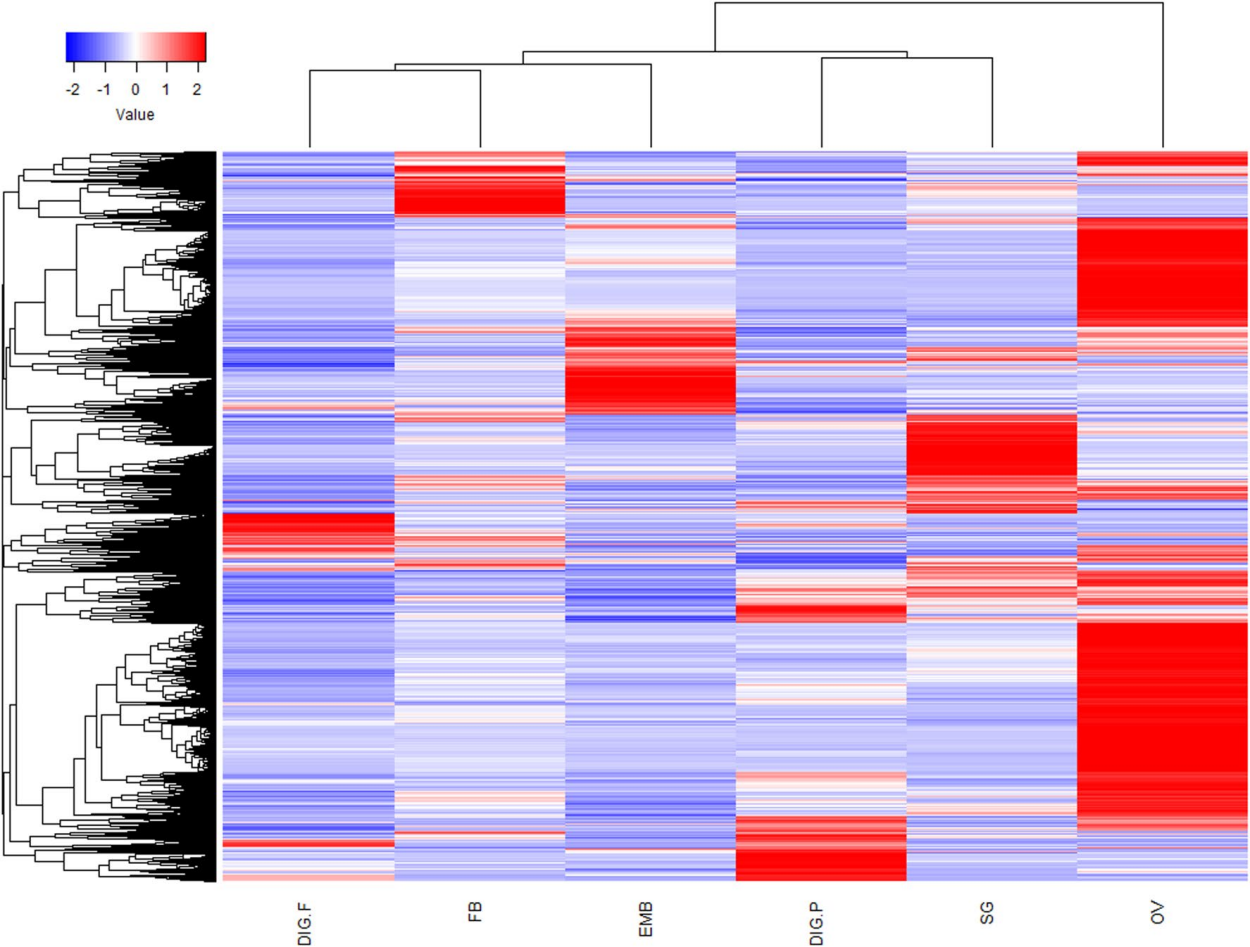
Differential expression of *Rhipicephalus microplus* transcriptome. Heatmap of normalized RPKM data from embryos from 1, 3, 5, 7, 9, 11, 13 day-old eggs (EMB), ovaries from partially and fully engorged adult females (OV), salivary glands from partially engorged adult females (SG), fat bodies from partially and fully engorged adult females (FB), digestive cells from partially engorged adult females (DIG.P), and digestive cells from fully engorged adult females (DIG.E). The color scale “Value” accounts for the Z-score deviation from the mean by standard deviation units.

Reads from each library were mapped back into the assembled transcriptome and provided a view of gene expression in different tissues (Figs. 1, 2, and Table S2). Although reads from the synganglion were used for assembly, data from this tissue were not used for expression analysis in this study. Functional classification of the transcriptome by total number of CDS or according to normalized RPKM values for each class (Figs. 1, 2, and Tables S1–S8) highlighted the complexity of the resulting assembly and helped to visualize the significant metabolic alterations that occur in these tissues during feeding. Accordingly, the secreted class contained 17% of the total contigs and normalized reads per kilobase per million (RPKM). CDS without a significant match in all databases (referred to as unknown—UK—in Table S1) and transcripts with a significant match in at least one database, but that code a protein without a known function (referred to as unknown conserved—UC—in Table S1), were classified simply as unknown; this class contained 29.5% of the CDS and 35.1% of total normalized RKPM (Table 3).

To visualize the full breadth of differential expression between tissues and time points, a heatmap was constructed using normalized RPKM values for CDS with an average of total RPKM from all tissues ≥ 1 (considering the reads of all libraries), in order to avoid inclusion of poorly expressed contigs, totaling 16,240 CDS. The data presented a characteristic transcriptional pattern for each tissue, revealing a clear tissue-specific clustering of overexpressed genes in all samples (Fig. 2).

### The cattle tick ovary and embryo transcriptome

#### Ovary, oogenesis and vitellogenesis

Embryo development of oviparous organisms relies on several different compounds, such as proteins, lipids, sugars and other molecules stored in yolk granules^24^. Oogenesis and vitellogenesis are essential processes for tick reproduction, including production of protein precursors and other molecules which are transported and accumulated inside the oocytes to ensure the embryo development^25^.

Data presented on a heatmap (Fig. 2) reveal the ovary as the tissue with a higher number of differentially expressed contigs (lower and upper right corner of the heatmap). To get a better insight on genes that are highly expressed in tick ovary, a total of 1091 transcripts, that are five times more expressed in ovary than in all combined tissues, were identified and categorized according to their putative function (Table S2). Transcripts coding for secreted proteins represent almost 17.4% of the total normalized RPKM in the ovary transcriptome. This functional class includes (i) proteins related to detoxification such as superoxide dismutase and arylsulfatase B, (ii) proteins with antimicrobial properties such as microplusin and ixoderin, (iii) proteases, (iv) proteins involved in signal transduction, and (v) proteins with unknown functions (Table S2).

Several transcripts coding for proteins with a well-known physiological role in oogenesis and vitellogenesis were identified in this study, including the predictable finding of several vitellogenin (Vg) transcripts (Rm-2788, Rm-23965, Rm-23966, Rm-23967, Rm-12079 and Rm-72548), which were highly expressed in fat body and in gut digestive cells^26^. This finding is consistent with the previous identification of the gut as an accessory site of Vg synthesis in ticks, in contrast to insects where Vg is mainly synthesized by the fat body. After being secreted to the hemolymph, the circulating Vg is uptaken by the growing oocytes, where it is named as vitellin (Vt) to highlight that some alterations are introduced in this storage lipoprotein after endocytosis. Vt is a major source of raw materials that support embryo growth. Proteases involved in Vt digestion in *R. microplus* have been described^25,27–33^. Interestingly, these enzyme transcripts are not abundant in ovaries, but are highly expressed in gut cells and fat body (Table S1, Tables S5–S8). This occurs because the ovary uptakes proteins synthesized and secreted in the hemolymph by other tissues. This phenomenon is suggested by data found here and is in accordance with previous studies^25,31,32,34^.

Transcripts coding for proteases involved in Vt digestion are overexpressed, such as the vitellogenin-degrading cysteine endopeptidase (VTDCE—Rm-15854), which was not significantly found in the ovary, and was overexpressed in DIG-F and FB (Table S1). The VTDCE synthesis by the gut and fat body, followed by protein export through hemolymph to the ovary was previously described^25^. Other examples are the tick heme-binding aspartic proteinase (THAP—Rm-14882) and *Boophilus* yolk-pro-cathepsin (BYC—Rm-849). From all the cathepsin-B coding transcripts with a role in oogenesis, only one (Rm-23945) is overexpressed in the ovary when compared with other tissues, suggesting a role in Vt degradation. A distinct pattern is observed for the Vg receptor (VgR—Rm-80856), since its transcript was expressed mostly in the ovary. The VgR is responsible for Vg uptake; the main reserve protein stored in oocytes for embryo development^35^.

Transcripts encoding for proteins involved in nuclear regulation and export from the nucleus such as cell division control protein, chromatin assembly and modification-related proteins, enzymes involved in DNA synthesis and repair, and histone-related proteins represent about 18% of total RPKM for the ovary. Similarly, proteins involved in signal transduction correspond to about 6% of total RPKM (Table S2), including enzymes such as acetylcholinesterase, juvenile hormone acid methyl transferase-like, kinases as serine/threonine-protein kinase and cyclin-dependent protein kinase, and amidases (Table S1). To date, juvenile hormones similar to those found in insects were not found in ticks^29,36^. However, a sequence coding to a transcript that is highly homologous to a juvenile hormone acid methyltransferase is overexpressed in the ovary (Rm-49336).

**Figure 3:**
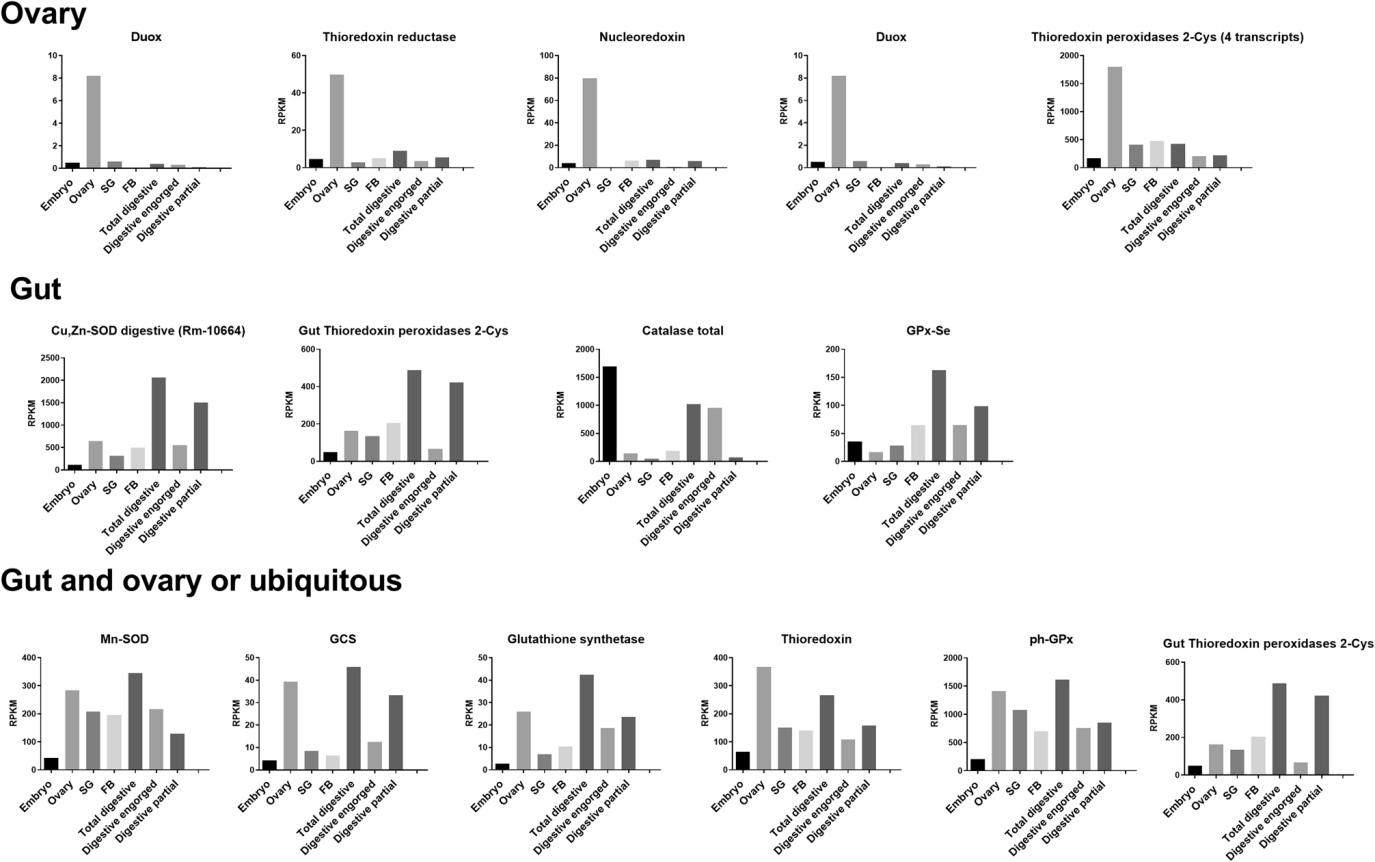
Expression analysis in the transcriptome of *Rhipicephalus microplus.* The graphs represents the antioxidant genes expression by each tissue*.* SG (salivary glands from partially engorged adult females) and FB (fat bodies from partially and fully engorged adult females). Transcript levels are expressed as RPKM.

The analysis of expression of the detoxification genes (Fig. 3) in all organs revealed a specific profile for ROS metabolism in the tick ovary. Out of the 13 transcripts coding for cytosolic Cu, Zn superoxide dismutases (SODs), 12 showed a marked overexpression in the ovary. A similar profile was found for two (out of four) thioredoxin reductase transcripts, four thioredoxin peroxidases (out of five transcripts for this enzymatic activity), and one nucleoredoxin. Although highly expressed also in digestive cells, gamma-glutamyl cysteine synthetase and glutathione synthetase, together with thioredoxin and a phospholipid-dependent glutathione peroxidase, showed high expression in the ovary as well (Fig. 3). This suggests, that among all tick tissues analyzed in this study , the ovary presents an especially robust redox metabolism; the major effectors would be the Cu, Zn-SODs, thioredoxin, thioredoxin peroxidases, and a phospholipid hydroperoxide glutathione peroxidase, fueled by reductive potential provided by a glutathione/thioredoxin couple, which is two pools of energy bridged by thioredoxin reductase.

The presence of a robust flow of antioxidant potential calls for an equally high source of reactive oxygen species. The fact that two transcripts coding for Duox type NADPH oxidases also have a predominant expression in ovaries make these proteins a candidate for this role. Interestingly, this enzyme has been ascribed a role as the source of hydrogen peroxide for chorion protein cross-linking in an insect^37^, a role that could well be assigned to these tick homologs.

Duox enzymes also have been ascribed a central role in the control of gut microbial symbionts in several insect species^38,39^. In ticks, the ovary is the residence of intracellular bacteria symbionts that are essential to tick development^40–42^. However, Guizzo et al.^43^ recently reported a quantitative analysis of the *R. microplus* microbial community revealing that, different from most animals, the tick gut harbors a very modest (if any) resident microbiota, in contrast to the ovary which has a large population of a *Coxiella* mutualist symbiont, that increase following the blood meal, paralleling the course of ovarian growth^43^. Interesting, *Amblyomma sculptum* and *Amblyomma aureolatum* have distinct transcriptional profiles of gut^44^ and microbiota composition^45^ that can be related to susceptibility to *Rickettsia rickettsii*.

Therefore, one possibility is that regulation of the expansion of the *R. microplus* microbial population in the ovary might also involve production of reactive oxygen species, making it essential for a panel of antioxidants to maintain intracellular redox homeostasis. This last hypothesis is in accordance with the identification of seventeen transcripts showing similarity to the antimicrobial peptide microplusin (Table S9). Levels of microplusin mRNA gradually increase along ovary development, reaching an impressive highest value three days after the adult females have dropped from the hots and start oviposition^46^. Moreover, microplusin is also present in hemolymph^47^. Importantly, it was recently reported that microplusin plays a role in protecting the tick *Amblyomma aureolatum* from infection with the tick-borne *Rickettsia rickettsii*^48^.

Research on insect reproduction has characterized many physiological and molecular properties of ovary and egg development^49^. By contrast, much less is known about these mechanisms in ticks^19,50^. In the ovary, this process is characterized by intensive cell division that determines oocyte and follicle cell development, and lately, the egg number. In this context, cell cycle regulation is well conserved in all organisms from yeast to humans^51^. Protein kinases participate in the regulation of cell division cycle controlling different pathways in this process. Cyclin-dependent kinase (CDK), aurora kinases, polo-like kinases, and checkpoint kinases (Bub1, BubR1, and Mps1) are the most important protein kinases related to cell division. Figure S1 shows the abundance of total CDK contigs by tissue. As expected, based on its function, the ovary has the highest abundance (55%) of CDK contigs. The CDKs 1, 5, and 10 are the most abundant sequences, while cyclin A-coding contigs represent the major cyclin observed in tick tissues, including ovaries (Figure S1). CDKs were previously characterized in *R. microplus* and *I. persulcatus* ticks^52,53^. Yet regarding control of cell fate and development, nucleoredoxin, a member of the thioredoxin family known to regulate the Wnt/beta-catenin signaling pathway in a redox-dependent way, also showed a high expression level in the ovary^54^.

#### Embryogenesis

Comparative analysis of embryo allowed the identification of 347 transcripts that are five times overexpressed in embryos than in other analyzed samples (Table S3). Interestingly, genes expressed in the embryo frequently were not five times more expressed than the sum of the other analyzed samples, possibly as a consequence that in embryo several different genes in diverse tissues and organs are being simultaneously expressed. Concerning the metabolism, 90 transcripts involved on metabolism of carbohydrates, lipids, and amino acids are transcribed in embryos. Among them, 25 transcripts are highly abundant, including enzymes of glucose metabolism; mainly glycolysis, gluconeogenesis, and pentose phosphate pathway. Interestingly, these genes were not highly expressed in ovaries, suggesting that these genes are transcribed during embryogenesis, and the mRNA or proteins are not maternally inherited.

Among these 347 embryo-specific transcripts, those encoding transposons, lipocalins, glycine-rich proteins, and secreted proteins are the most abundant. These secreted proteins are homologous to genes previously described as defensins or genes involved in immune response. Some of these secreted molecules specifically expressed during embryogenesis could be trans-peptides involved in immune response during embryogenesis. Such a finding would be important for embryo survival, since chelicerates such as ticks do not display an extraembryonic enveloping protective layer such as the serosa, which is involved in embryonic immune response in insect egg. Conversely, protective layers have independently evolved in other chelicerates such as scorpions^55–57^. In insects, these protective cellular layers prevent the loss of water and secrete defensins against bacterial infections; these protective layers are absent in tick embryonic development. These secreted proteins may have a role in immune protection during the long embryogenesis of *R. microplus,* which takes about 21–22 days in a potentially harmful environment ^58^.

### The cattle tick sialotranscriptome

As a blood-feeding parasite, *R. microplus* saliva is a complex mixture rich in bioactive compounds that modulate host hemostasis and immunological defenses to allow tick feeding activity^59,60^. In recent decades, cumulative information from transcriptomic and proteomic analyses of salivary glands and tick saliva of several tick species have provided insights into the immunological interactions at the tick–host interface. In addition to facilitating blood-feeding, the antihemostatic and immunomodulatory activities of tick saliva may also support survival and establishment of hemopathogens in the host^61^. *R. microplus* larvae attach to its host and then begin to feed and molt in nymphs before molting into immature adults, a process that takes around 12 days^59^. After mating, adult partially engorged females take larger blood meals to complete the maturation within 21–22 days and detach^59,62^. In the current study, SG were dissected from adult females feed on the host during 17 and 22 days. Therefore, data shown here combine part of the slow feeding phase and of the final rapid feeding phase. Consistent with reports that other tick species change salivary composition during feeding^63–67^, data in this study reveal that 320 transcripts are overexpressed in SG when compared to other tissues, including contigs encoding metalloproteases, proteinase inhibitors, and lipocalins (Table S1 and Table S4).

Tick metalloproteases have been suggested as participating in platelet disaggregation and blood coagulation by fibrinolytic and gelatinase activities, thereby facilitating blood feeding^68^. The observation that members of family M12 metalloproteases, similar to hemorrhagic proteases of snake venom, are overexpressed in the SG of *R. microplus*, suggest they have a role of this class of protein in tick feeding physiology. Snake venom M12 proteases were associated with hemorrhaging, edema, hypotension, hypovolemia, inflammation, and necrosis^69^. *I. scapularis* saliva has a specific metalloprotease similar to the hemorrhagic protease of snake venoms displaying gelatinase and fibrin(ogen)olytic activities^68^. In a related study, RNAi silencing of metalloproteases from *I. ricinus* impaired blood meal feeding and egg laying^70^. In *R. microplus*, vaccination with recombinant metalloprotease 4 (rBrRm‐MP4) significantly decreases the number of ticks that complete the life cycle on the host and egg hatching, providing an overall protection of 60% against tick infestation^65^.

Protease inhibitors, including Kunitz, TIL (trypsin inhibitor-like), and cystatin were overexpressed in SG (Table S1 and Table S4). Most Kunitz protease inhibitors target serine proteases, although some also block ion channels^71^. In ticks, some protease inhibitors have several Kunitz domains *in tandem*, and they are classified as monolaris, bilaris, trilaris, and so on, according the number of Kunitz domains. In *R. microplus*, 11 Kunitz protease inhibitors are overexpressed in the salivary glands, including nine monolaris and two bilaris (Table S1). Several members of Kunitz family have been functionally characterized as anti-blood clotting agents such as savignin^72^, the antithrombin boophilin^73^, as well as ixolaris, an anticoagulant that binds both factor VIIa and factor X^74^. Interestingly, in *R. microplus* transcriptome these contigs encoding Kunitz proteins are overexpressed mostly in salivary gland, as expected, but also showed high expression levels in digestive cells, which is in accordance with inhibition of blood clotting being supported not only by saliva but also being reinforced after blood ingestion by secretion by digestive cells. Cystatins are ubiquitous, reversible, and tight-binding inhibitors of cysteine proteases. Some tick salivary cystatins have immunosuppressive and anti-inflammatory activities^75^. A total of 27 cystatin coding sequences were identified in this study, and one contig is overexpressed in salivary glands. Proteins containing TIL (trypsin inhibitor-like) signature domains have been reported in blood-feeding mosquitoes and tick sialomes^76^. In *R. microplus*, TIL family members include peptides closely related to tick elastase inhibitors, which also have antimicrobial activity^77^, with a total of three contigs overexpressed in SG (Table S1 and Table S4).

Lipocalins are single modular proteins of around 200 amino acids that fold tightly in a β-barrel with potential for binding small hydrophobic molecules in a central pocket. Annotation of the most recently identified tick lipocalins is based on homology with annotated histamine-binding proteins from other tick species, displaying the characteristic PFAM tick histamine-binding domain (PF02098)^78^. Contigs matching the tick histamine-binding domain were identified being overexpressed in SG of *R. microplus* (Table S1 and Table S4). The high abundance of lipocalins was also confirmed in the proteome of *R. microplus* saliva^60^. Some of these *R. microplus* identified lipocalins have similarities with some lipocalins described in other ticks, which have antihemostatic and immunomodulatory activities^79^ such as the capacity to bind biogenic amines, which is compatible with tick saliva anti-hemostatic and immunomodulatory roles during blood feeding.

### Digestive cells

A unique feature of tick physiology that differs from hematophagous insects is that blood digestion is entirely intracellular, occurring inside a differentiated cell lineage: the so-called digest cells (DIG)^80^. After erythrocyte lysis, blood proteins are taken up by endocytosis and digested in acidic vesicles by lysosomal hydrolases^81^. To address this singularity of the biology of ticks, we prepared cDNA libraries from DIG that were isolated from (1) partially engorged adult females (DIG-P) that were still engaged in the process blood feeding and were in the “big sip” stage, and (2) fully engorged females (DIG-F) that had already completed blood meal and have detached from the host. To our knowledge, this is the first transcriptomic analysis of this cell type, as other previous studies on gene expression in tick gut have used whole organ extracts^16,82,83^.

Taken together, the analysis of these libraries showed 634 sequences that are overexpressed in DIGs (Table S6). From those, only three specific groups of functionally assigned transcripts accounted for more than 70% of the RPKMs from the most overexpressed contigs, namely secreted (61%), immunity-related (5%), and unknown conserved (6%). This marked departure from the class distribution profile obtained when all libraries are pooled together is probably a consequence of the unique physiology of this cell type exclusively found in ticks. Interestingly in DIG-P (Table S7), the most abundant contigs are classified as secreted (78.5%), unknown (12.1%), unknown conserved (3.2%), and immunity-related (2.4%). However, this profile changes to secreted (46.1%), oxidant/detoxification metabolism (14.3%), protease inhibitor (12.6%), and immunity-related (11.4%) in DIG-F (Table S8), possibly reflecting the alteration in metabolism and functions of the DIG, which must deal with a pro-oxidant menace represented by hemoglobin proteolysis and heme release. The most highly expressed transcript (Rm-24035) in this group is homologous to the second most highly expressed transcript in the transcriptome of the *Dermacentor variabilis* gut^84^ and accounts alone for close to 20% of the total digestive secretome RPKM of *R. microplus* (Table S1, Tables S6–S8).

Vertebrate blood is ~ 85% protein in dry weight, and therefore proteases are expected to be highly expressed in DIG. As a consequence of the intracellular mode of digestion of the blood meal, proteases found highly expressed in the transcriptome are acid lysosomal hydrolases such as legumains, cysteine, and aspartic proteinases, coherently with previous reports^80,85,86^. Notably, among all tissues analyzed here, the most highly expressed proteases (with signal peptide, suggesting that targets the protein to a secretory pathway) were found in the DIGs libraries. Noticeably, although several inhibitors of cysteine proteinase (cystatins) were identified, these were nearly ten times less expressed than inhibitors of serine proteases. These inhibitors can be related to the inhibition of mammalian proteases involved in blood coagulation and complement cascades^87^, acting as a regulator of blood digestion in gut^88^ and modulation of the tick immune proteolytic cascades, that have been ascribed relevant roles in the interaction with the microbiota and with pathogens^89^. However, it is also possible to speculate that part of these inhibitors might be negative regulators of hemoglobin degradation, as the proteolytic activity involves the release of large amounts of heme, a pro-oxidant molecule^90^. Keeping the pace of hemoglobin degradation with heme detoxification should be of paramount importance for this cell. Regarding this topic, it is relevant to observe that in DIGs a significant expression of three transcripts coding for an aspartic proteinase, whose activity was shown to be inhibited by heme (THAP, for tick heme-binding aspartic proteinase), was previously described as related to oogenesis^28^. In this study, THAP was found almost exclusively in the gut (97% of the RPKM); a result that makes it very likely that THAP may also play a relevant role in dietary hemoglobin degradation. However, we also identified the presence of two other contigs of probable paralogous enzymes (Rm-14881 and Rm-14882, Table S1) that are predominantly expressed in the fat body, which might code for the protein originally found in eggs and for this reason ascribed a role only in yolk degradation^28,32^.

**Figure 4:**
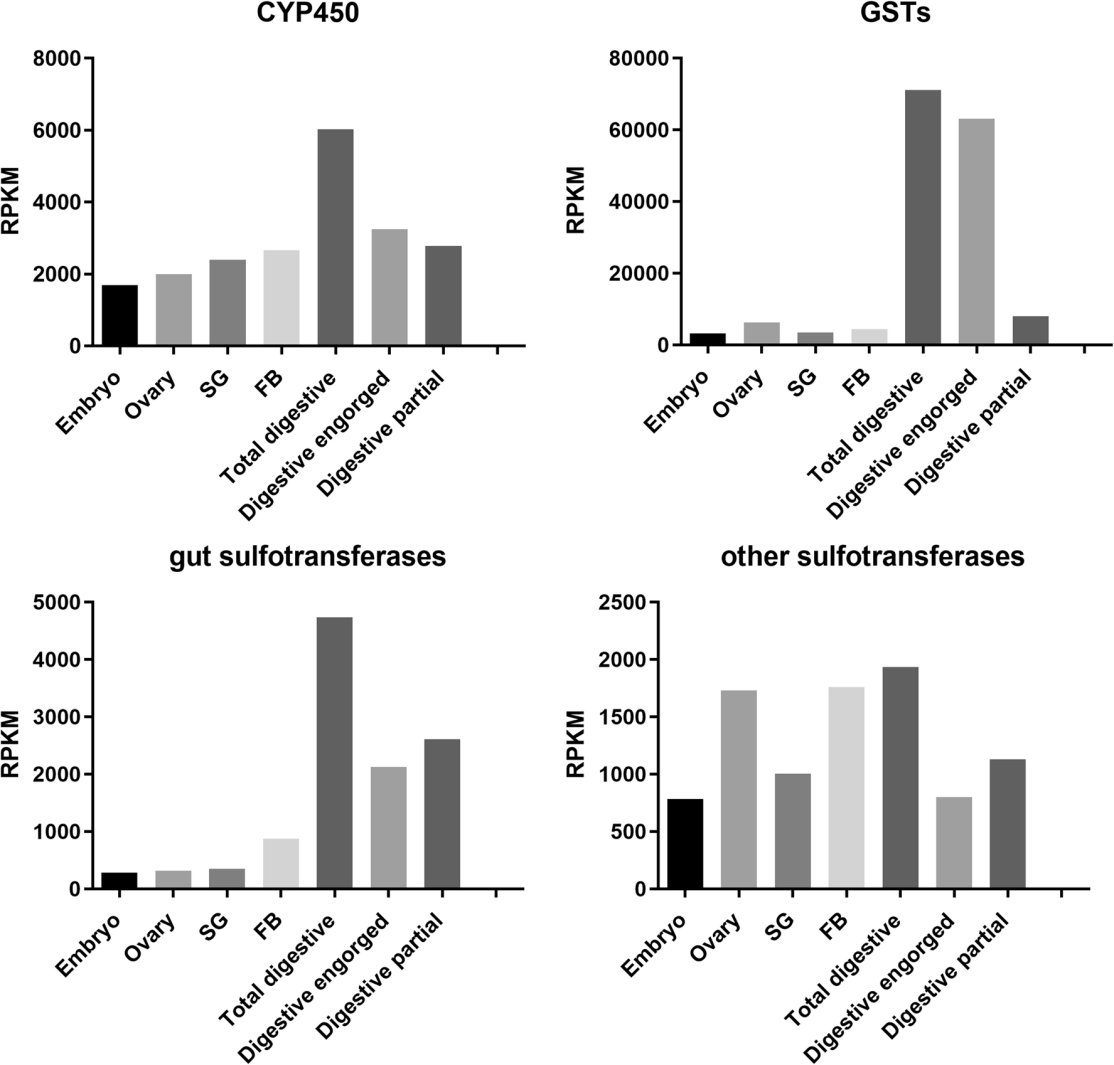
Expression analysis in the transcriptome of *Rhipicephalus microplus.* The graphs represents the expression of detoxification-related genes in each tissue*.* SG (salivary glands from partially engorged adult females) and FB (fat bodies from partially and fully engorged adult females). Transcript levels are expressed as RPKM.

The detoxification-related functional class is one of the most overexpressed class in DIGs (Table S1 and Tables S6–S8). For most hematophagous insects, it is assumed that the main dietary pro-oxidant component is the abundant heme and iron from the vertebrate blood, but transcriptomes of mosquitoes, triatomine bugs, and other tick species also showed high expression levels of cytochrome P-450 (CYP 450) and glutathione S-transferases (GSTs)^64,91,92^. These classical phase I and phase II detoxification enzymes belong to large gene families with multiple copies in the genome of insects and ticks, having essential roles in different pathways, especially in lipid metabolism. Taken all together, the 121 CYP 450s transcripts show a high RPKM value in all tissues analyzed here, including the DIG libraries. Several individual transcripts, however, showed a very tissue-specific expression pattern, including 29 CYP 450 transcripts that are at least fivefold more expressed in the DIG than in all other libraries taken together (Tables S6–S8). This finding highlights that each tissue needs a CYP 450 composition fitted to its own metabolic demands (Fig. 4A). In contrast, 80% of the total RPKM for all 59 GST transcripts are expressed in the DIG libraries, particularly in DIG from fully engorged females (Fig. 4B). This is largely due to only two GST transcripts that are members of the N delta epsilon subfamily, are very highly expressed in the digestive cells (Rm-18369 and Rm-2328), and show increased expression after the blood meal. Similarly, two GST transcripts were found in the gut and ovaries of the tick *D. variabilis,* and they were up-regulated in *Rickettsia*-infected and upregulated upon tick feeding^93^. Interestingly, a microarray analysis of the effect of *Babesia bovis* infection on whole gut gene expression^83^ identified one CYP 450 as the most highly upregulated transcript and a GST as one of the down-regulated genes.

A putative secreted ferritin (Rm-68449) was highly transcribed in DIGs, similar to *I. ricinus* Fer 2, which knockdown leads to high tick lethality^94^, revealing an essential role in the absorption and transport of iron. It was previously shown that ticks do not synthesize heme^95^ and also do not have heme oxygenase, which cleaves the heme porphyrin ring and releases the iron atom in most organisms, rendering it available. As a consequence, ticks are not able to obtain iron from heme breakdown, relying solely on dietary iron to make their essential ferroproteins^96^, highlighting the central role played by this secreted ferritin in the tick life cycle.

Analysis of transcripts coding for classical antioxidant enzymes showed a common core of antioxidant potential sustained by glutathione synthesis (GCS, GS), thioredoxin, and two transcripts coding for thioredoxin reductases, a phospholipid-dependent glutathione peroxidase, and the mitochondrial Mn-SOD that are expressed in all tissues. DIG libraries, however, showed particularly high expression levels of specific isoforms for the cytosolic Cu, Zn SOD, and a thioredoxin peroxidase (2-cys family), plus a selenium glutathione peroxidase and catalase, highlighting a focus on hydrogen peroxide detoxification by this cell type. A result that is in line with previous experimental data reporting the severe effect of chemical inhibition of catalase on blood fed ticks^97^.

Another class of enzymes that appeared here highly expressed but are usually not particularly highly expressed in the gut of insect vectors, are sulfotransferases. They were included here in the general class of detoxification, because adding sulfate from a high-energy donor is a detoxification mechanism in some cells. On the other hand, one of the products of the reaction catalyzed by this enzyme, 3′-phosphoadenosine-5′-phosphosulfate (PAP) is a sulfate donor to the biosynthesis of sulfated polysaccharides, major components of extracellular matrix of the tick gut^98^. This hypothesis is consistent with some sulfotransferases (such as Rm-25779, Rm-18149, Rm-64114, and Rm-139775) decreased expression in digestive cells after blood meal, making it likely that those enzymes are acting as players in extracellular matrix production that might support the growth of cells (Fig. 4C,D). Sulfotransferases are also involved in the inactivation of octopamine^99^, which functions as a neuromuscular transmitter in insects^100^.

A major feature of tick gut DIGs is the presence of an organelle dedicated to heme detoxification, the hemosome. A heme transporting pathway directs heme derived from hemoglobin degradation in the acidic digestive vacuoles to the cytosol and then to the hemosome. Several ABC transporters have high expression levels in DIGs and therefore are likely candidates to function as heme transporters, including Rm-73791 that is almost exclusively expressed in DIGs. This is in accordance with previous biochemical data^101^, where ABC-like transporters were implicated on heme transport in digestive cells of *R. microplus*. In addition, although this transporter has considerable expression levels in gut, it is even more intensively expressed in the ovary, which raises the interesting possibility that these transporters may be involved in heme transport to the growing oocyte, where vitellin is a major heme-storage protein.

Among typical immune effectors, only defensins seem to be highly expressed in the DIG. However, transcripts receiving an immune label were mainly related to bacterial killing by phagocytosis (ixoderins, plus a set of proteins related to lysosomal function). This is consistent with the digestive function of these cells being exerted by means of digestion of blood proteins in a lysosomal acid compartment and can be related to very low bacterial levels observed in the gut, including during blood feeding^43,102^.

### The cattle tick energy metabolism

Considering the relatively low amount of glucose ingested by the cattle tick, since the concentration in blood is around 50 mg/dL^103^, carbohydrate metabolism in this organism deserves some discussion concerning the correlation with this very rich protein diet. Two hexokinase coding transcripts were found in the six libraries analyzed. The isoform represented by Rm-54671, is almost 30 times more expressed than Rm-19014, but it is from six to seventy times less expressed in digestive cells than in any other tissue (Table S1 and Table S6). Interestingly, besides the role played in metabolism, hexokinases are also enrolled in the inhibition of the intrinsic pathway apoptosis, preventing the release of cytochrome *c* from the mitochondria^104^. Therefore, it is possible that hexokinases are also involved in controlling the apoptosis in *R. microplus* organs.

The essential enzymes needed to the synthesis and degradation of glycogen are present in this dataset. The sequences of phosphoglucomutase (Rm-32898), UDPG-pyrophosphorylase (Rm-37883), UTP-glucose-1-phosphate uridyltransferase (Rm-26034), glycogen synthase (Rm-18733), and glycogen phosphorylase (Rm-26011) are observed in all tissues. The enzymes involved in glycogen accumulation are poorly expressed in digestive cells of fully engorged females. The transcript coding for hexokinase, the first enzyme of the glycolytic pathway, has a very low expression in digestive cells of both partially and fully engorged females; we could not find a transcript coding for phosphofructokinase-1. In contrast, the glucose 6-phosphate-phosphatase transcript was highly expressed, especially in digestive cells of partially engorged females. The high abundance of this transcript in digestive cells led us to speculate that most of the glucose in these cells might not come from the blood meal but from gluconeogenesis, and later being exported to hemolymph and other tissues. The following six enzymes catalyze reactions that are reversible in cellular conditions; the direction of these reactions thus being governed by the law of mass action, and therefore participating both in glycolysis and gluconeogenesis: fructose-bisphosphate aldolase (Rm-1568), triosephosphate isomerase (Rm-4663), glyceraldehyde 3-phosphate dehydrogenase (Rm-10107), 3-phosphoglycerate kinase (Rm-26739), phosphoglycerate mutase (Rm-62495), and enolase (Rm-19039) were found. Their expression levels, although varying among organs, are higher than those of transcripts coding for enzymes that are participating only in glycolysis but not in gluconeogenesis (Table S1).

All subunits of pyruvate dehydrogenase were found: Rm-169115 is E1 alpha subunit; Rm-8313 is E1 beta subunit; Rm-56844 is dihydrolipoamide dehydrogenase; and Rm-12428 is dihydrolipoamide acetyltransferase. The tricarboxylic acid cycle is a central metabolic pathway. All transcripts coding enzymes and their subunits were identified, and the expression of these enzymes shows differences among different tissues; the TCA cycle appearing to be more active on the salivary glands, ovaries, and digestive cells of partially engorged females. Carnitine O-palmitoyltransferases I and II ORF, involved in fatty acids oxidation metabolism (Rm-12481 and Rm-18519, respectively), were expressed in all organs tested. Nevertheless, their expression is higher in salivary glands. Moreover, ORFs of all enzymes necessary for β-oxidation were found, showing that conversion of fatty acids into ketone bodies occurs in *R. microplus* (Table S1).

Enzymes coding for the pentose phosphate pathway are all present in all tissues but are particularly highly expressed in the ovary. This could account for the high demand for NADPH reducing power by the glutathione/thioredoxin dependent antioxidant mechanisms described above, as well as by the very pronounced concentration of the lipid biosynthesis in the ovary (fatty acid synthase transcripts Rm-43199, Rm-25088, and Rm-25089, and two for acetyl CoA carboxylase Rm-44844 and Rm-44843).

### The cattle tick immune system

The transcriptome of *R. microplus* revealed 210 CDS classified in the immune system category (Table S1). Among them, important components of immune signaling pathways, antimicrobial peptides, and recognition proteins were identified. Additional CDS of proteins that belong to other functional categories, such as nuclear, protease and protease inhibitors, detoxification, and protein export and modification, but that still play a role in immune responses, were also identified (Table S9). Importantly, 43 of the 210 sequences in the immune system category were overexpressed (fold-change ≥ 5) in one specific organ of the tick (Table S9). Specifically, 24 were overexpressed in DIG, including one defensin (but not lysozymes nor microplusins, that were expressed in other tissues) and showing an immune profile dominated by ixoderins (fibrinogen-related proteins with lectin activity, involved in microorganisms phagocytosis, 9 transcripts)^105,106^, ML [(MD-2 (myeloid differentiation factor-2)-related Lipid-recognition)] domain containing proteins (involved in lipid trafficking and lysosomal function, 5 transcripts), and GILT (gamma-interferon inducible lysosomal thiol reductases, and 4 transcripts)^107,108^. GILT are the only molecules capable of keeping essential SH residues of cysteine proteinases reduced at acid pH, therefore preserving their activity inside the endosomal pathway^109^. As mentioned above, the *R. microplus* gut is inhabited by a very minute microbial population, probably reflecting the operation of a powerful microbe killing control, such as the production of hemocidins^110,111^. Data presented here suggest that the endosomal/lysosomal pathway also play a central role in the gut immunity of ticks (Table S9), which differs from the mode of operation of the insect gut. Indeed, it was previously reported that the immune responses of the tick gut is involved in delineating susceptibility to the zoonotic pathogen *R. rickettsii*^44^. While rickettsial infection downregulates immune-related genes in the gut of *A. aureolatum*, immune-related genes are upregulated in *Amblyomma sculptum*, which is much less susceptible to infection with *R. rickettsii* than *A. aureolatum*^48^. Interestingly, most of the immune-related transcripts show a very different pattern of expression between libraries from fully engorged and partially engorged ticks, suggesting the existence of different immune challenges between these two phases in the tick life cycle. A more detailed account of the immune-related transcripts found in this work follows.

#### Immune signaling pathways

Different from the vastly available information on the immune signaling pathways in insects, very little is known about this subject in ticks. Sequences encoding components of immune signaling pathways were identified in the genome of *Ixodes scapularis*^9,112,113^. A study on *R. microplus* immune signaling pathway components revealed that Toll and JAK/STAT pathways of ticks are closely related to those of insects and crustaceans^114^. Conversely, certain components of IMD pathway of apomorphic orders of insects (Diptera and Coleoptera) are missing in ticks, as well as in plesiomorphic orders of insects (Hemiptera) and crustaceans. However, it was observed that IMD pathway is functional in *I. scapularis*^115^ and in *R. microplus*^116^ , although little is known about its activation and effectors. Reports on functional characterization of components of tick immune signaling pathways are also scarce. It has been suggested that the JAK/STAT pathway plays a role in the control of infection by both *Anaplasma phagocytophilum*^117,118^ and *Borrelia burgdorferi*^119^ in *I. scapularis*. Moreover, it was reported that the host IFNγ upregulates a tick GTPase through the JAK/STAT pathway, limiting *B. burgdorferi* proliferation^120^. The IMD pathway was also enrolled in protection of ticks against infection. The knockdown of the transcription factor Relish increased the susceptibility of *R. microplus* to *Anaplasma marginale*^116^ and of *I. scapularis* to *A. phagocytophilum*^115^. Interestingly, it was shown that the activation of IMD pathway does not occur through recognition of bacterial peptidoglycans by PGRPs (peptidoglycan-recognition proteins) as in insects, but through recognition of bacterial lipids^115^.

In the current study, we identified five CDS of Toll-like receptors and two CDS of the cytokine spaetzle, which are components of Toll pathway (Table S1). Other components of this same pathway, such as the adaptor MyD88 and cactin, the interactor protein of the NF-kB inhibitor cactus (IkB), were also identified (Table S1). Regarding components of IMD pathway, we identified the NF-κB relish (Rel/NF-κB) and caudal, its inhibitory protein (Table S1). In addition, transcripts of the inhibitor proteins PIAS (protein inhibitors of activated STAT) and SOCS (suppressor of cytokine signaling) of JAK/STAT pathway were also detected. In relation to the JNK pathway, we identified a CDS of the transcription factor Jun (Table S1). Notably, all components of immune pathway components identified in the present study have been previously identified^114^. This result reinforces the constitutive expression of signaling pathway components in *R. microplus*, as previously suggested^114^.

#### Antimicrobial peptides

AMPs are important effectors of immune signaling pathways and represent pivotal factors of invertebrate immune system, acting directly against a wide range of microorganisms, such as protozoa, bacteria, and fungi^121^. Among the AMPs described in ticks, defensins are a prominent group. These small peptides are synthesized as preprodefensins and are present in several species of both soft and hard ticks^122^. In *R. microplus*, one defensin was found in hemocytes^47^. Importantly, the knockdown of varisin, a defensin from the tick *D. variabilis*, reduced in 50% the activity of hemolymph against the Gram-positive bacterium *Micrococcus luteus*^123^. On the other hand, silencing of this same gene intriguingly reduced the infection of the tick-borne pathogen *A. marginale*^124^. Also, defensins were reported to be differentially expressed upon infection with *Rickettsia montanensis*^125^. In addition to hemolymph, expression of defensins was also reported to occur in other tick organs, for example the gut and salivary glands. Nineteen defensin CDS were here identified in the *R. microplus* transcriptome. Among them, one CDS exhibited high transcript levels in digestive cells and another in salivary glands (Table S1). Defensin transcripts, besides transcripts of other AMPs, were also previously identified in the salivary glands of other tick species^48,126^.

The presence of AMPs as well as other immune compounds in the tick gut could be relevant because the slow intracellular digestion of nutrients, as well as neutral pH, may favor the proliferation of microorganisms. In addition to defensins, lysozymes were reported to be present in the gut of ticks, especially in the soft tick *Ornithodorus moubata*^127,128^. We identified five CDS of lysozymes in the *R. microplus* transcriptome (Table S1) without overexpression in a specific tick organ, and none of them showing expressions levels comparable to defensins or microplusins (see below). Interestingly, in addition to the endogenous AMPs, peptides derived from host hemoglobin (hemocidins) were also detected in the tick gut^111^. Production of hemocidins occur by action of aspartic (cathepsin D-type) and cysteine (cathepsin L-type) peptidases. Notably, transcripts of both cathepsins D- and L-types were detected in the current study (Table S1).

Histidine-rich peptides were identified in hemolymph^47^, ovaries and eggs^46^ of *R. microplus* (microplusin) and in synganglion of *Amblyomma hebraeum*^129^. It was shown that microplusin has the property of chelating copper, inhibiting the respiration of both bacteria and fungi^130,131^. Twenty-one microplusin CDS were detected in the present study (Table S1). Three of them presented high transcript levels in the fat body and two in the ovary (Table S5 and Table S2). Infections can affect tick fertility, since resources can be diverted from reproductive process to the synthesis of immune factors and tissue repair. Indeed, it was previously shown that *R. rickettsii*-infection diminish the reproduction rate in ticks^132^. As mentioned above, it was recently reported that the knockdown of microplusin increases the susceptibility of *A. aureolatum* to infection with *R. rickettsii*^48^. However, microplusin showed no effect on either the bacterial transmission to the host or the tick fitness.

In conclusion, this study described the transcriptome assembly of various tissues of *R. microplus* and expanded the number of genes annotated for this economically important parasite. Functional annotation and classification revealed that many genes related to blood digestion and host-parasite interaction are activated in gut cells compared with other tissues. Furthermore, essential genes for cell development and embryogenesis were overexpressed in ovaries. A number of candidate genes for important biochemical pathways were also identified. The availability of this transcriptome opens new perspectives in the study of biochemical role of gene products. Finally, these novel data provide useful information on the tick physiology and serve as a valuable platform to support the study and development of new control methods.

## Material and methods

### Ethics statement

All animals used in these experiments were housed at Faculdade de Veterinária, Universidade Federal do Rio Grande do Sul (UFRGS). This study was conducted considering ethic and methodological aspects in agreement with the International and National Directives and Norms by the Animal Experimentation Ethics Committee of the Universidade Federal do Rio Grande do Sul (UFRGS). The protocol was approved by the Comissão de Ética no Uso de Animais (CEUA) – UFRGS (project 14403).

### Ticks

*Rhipicephalus microplus* ticks (Porto Alegre strain) free of *Babesia* sp. and *Anaplasma* sp. were maintained on Hereford bovines acquired from a naturally tick-free area (Santa Vitoria do Palmar, RS, Brazil). The bovines were housed in individual thick-proof pens on slatted floors and infested with 20,000 10-day-old *R. microplus* larvae per animal^59^. Partially engorged female (PEF) ticks were manually removed from cattle, while fully engorged female (FEF) ticks were obtained after spontaneous detachment from the host. After collection, ticks were washed with 70% ethanol and had the dorsal surface dissected with a scalpel blade.

### Isolation of digestive cells

Digestive cells were isolated as previously described^81^. Essentially, ticks were dissected in Petri dish containing sterile PBS, guts were isolated, opened gently with tweezers, and cells were detached from the gut wall manually with tweezers. Subsequently, cells were carefully collected using a 1-mL pipette tip, washed three times in sterile PBS and placed in a 12-well culture plate. In order to reduce the amount of debris and gut soluble contents, cells were washed with a gentle flow of culture medium (L-15 Leibowitz’s medium supplemented with 150 mM NaCl) produced by a 1-mL pipette tip. During the isolation of the digestive cells, the gut was dissected and kept in an ice-cold medium. The procedure can be observed by the movie file included as supplemental material (Supplementary video 1).

### RNA extraction, cDNA library construction and sequencing

Tissues (as described below) were separated with fine-tipped forceps and washed in ice-cold phosphate buffered saline pH 7.2 (PBS). For egg collection, FEF ticks were collected and incubated in Petry dishes at 28 °C and 85% relative humidity until oviposition. For embryos, eggs were kept during twelve days in disposable tubes under the same conditions as described above. Total RNA was extracted from the following tissues: (i) embryos (1, 3, 5, 7, 9, 11, 13 day-old eggs); (ii) ovaries from partially and fully engorged females; (iii) salivary glands from partially engorged females; (iv) fat bodies from partially and fully engorged females; (v and vi) digestive cells from partially and fully engorged females, using TRIZOL reagent according to the manufacturers’ instructions (Thermo Fisher Scientific, Waltham, MA, USA). A total amount of 10 µg RNA was used as input material for the library preparation. Sequencing libraries were generated using the IlluminaTruSeqTM RNA Sample Preparation Kit (Illumina, San Diego, USA) following the manufacturer’s recommendations.

### Transcriptome assembly and bioinformatics

Bioinformatic analyses were performed as described previously^14^. Illumina adaptor sequences and low-quality bases were removed from the raw reads. The quality-filtered sequence reads of each sample were pooled to generate a single transcriptome assembly of the *R. microplus*. Reads were assembled with Abyss 1.3.3 software (https://www.bcgsc.ca/resources/software/abyss)^133^ with various k values (from 25 to 95 at five unit intervals). Because the Abyss software tends to miss highly expressed contigs, we have also run the Trinity 2.0 assembler (https://trinityrnaseq.github.io/)^134^ on the raw data. The resulting assemblies were joined by an iterative BLAST and CAP3 assembler^14^.

Coding sequences (CDS) were extracted using an automated pipeline^14^, based on similarities to known proteins, or by obtaining coding sequences from the larger open reading frame (ORF) of the contigs containing a signal peptide identified by version 3.0 of the SignalP software (https://www.cbs.dtu.dk/services/SignalP-3.0/)^135^. A non-redundant set of the coding sequences and their protein sequences were mapped into a hyperlinked excel spreadsheet. Signal peptide, transmembrane domains, furin cleavage sites, and glycosylation sites were determined with software from the Center for Biological Sequence Analysis (https://www.cbs.dtu.dk/services/). The automated annotation of the proteins was based on matches to various databases, including Gene Ontology (https://geneontology.org/)^136^, Pfam (https://pfam.xfam.org/)^78^, Swissprot (https://www.ebi.ac.uk/uniprot/), KOG (https://mycocosm.jgi.doe.gov/help/kogbrowser.jsf)^137^, SMART (https://smart.embl-heidelberg.de/)^138^, Refseq-invertebrates, and sequences containing Acari [organism] protein sequences obtained from GenBank (https://www.ncbi.nlm.nih.gov/genbank/). The manual annotation was performed as detailed in previous article^14^.

To estimate the transcripts abundance, reads were mapped back into the CDS using BLASTn with a word size of 25 (-W 25 switch). The matches were used if they had less than two gaps and if their scores were equal to the best score. The resulting BLAST file was used to compile the number of reads each CDS received from each library, and to count the number of hits at each base of the CDS, allowing for the determination of the average CDS coverage, percent linear coverage, as well as maximum and minimum coverage. Mapping of the reads and RPKM values were included in an Excel spreadsheet.

Finally, tick genomes^11,13^ were used as reference to assess the assembly quality in terms of gene completeness using BUSCO v. 4.1.3 (https://busco.ezlab.org/)^23^.

## Supplementary information

Supplementary Table S1.

Supplementary Information.

Supplementary Video 1.

## Data Availability

Raw sequence reads were deposited in the NCBI Sequence Read Archive (Biosample SAMN02463642 and Bioproject PRJNA232001). Transcriptome Shotgun Assembly project has been deposited at DDBJ/EMBL/GenBank under the accession GHWJ00000000. The version described in this paper is the first version, GHWJ01000000.
