# Supplementary material for: A physiologic overview of the organ-specific transcriptome of the cattle tick Rhipicephalus microplus
Source: Sci Rep. 2020 Oct 26;10:18296. doi: 10.1038/s41598-020-75341-w (PMC7588415; doi:10.1038/s41598-020-75341-w)
Supplement: Supplementary file 2 — Supplementary Information. [file 41598_2020_75341_MOESM2_ESM.pdf]

A physiologic overview of the organ-specific transcriptome of the cattle tick *Rhipicephalus microplus*

Lucas Tirloni[1,2], Gloria Braz[3,11], Rodrigo Dutra Nunes[4,11], Ana Caroline Paiva Gandara[4], Larissa Rezende Vieira[3], Teresa Cristina Assumpcao[12], Gabriela Alves Sabadin[1], Renato Martins da Silva[5], Melina Garcia Guizzo[4], Josias Alves Machado[5], Evenilton Pessoa Costa[5], Daniele Santos[5], Helga Fernandes Gomes[5], Jorge Moraes[5,11], Maria Beatriz dos Santos Mota[3], Rafael Dias Mesquita[3], Milane de Souza Leite[4,11], Patricia Hessab Alvarenga[6,11], Flavio Alves Lara[7,11], Adriana Seixas[9,11], Rodrigo Nunes da Fonseca[5,11], Andrea C. Fogaça[10,11], Carlos Logullo[5,11], Aparecida Sadae Tanaka[8,11], Sirlei Daffre[10,11], Pedro L. Oliveira[4,11], Itabajara da Silva Vaz Jr.*[1,11], José M. C. Ribeiro[12]

[1]Centro de Biotecnologia, Universidade Federal do Rio Grande do Sul, Porto Alegre, Brazil

[2]Tick-Pathogen Transmission Unit, Laboratory of Bacteriology, National Institute of Allergy and Infectious Disease, Hamilton, United States

[3]Departamento de Bioquímica, Instituto de Química, Universidade Federal do Rio de Janeiro, Rio de Janeiro, Brazil

[4]Laboratório de Bioquímica de Artrópodes Hematófagos, Instituto de Bioquímica Médica, Universidade Federal do Rio de Janeiro, Rio de Janeiro, Brazil

[5]Instituto de Biodiversidade e Sustentabilidade NUPEM, Universidade Federal do Rio de Janeiro, Rio de Janeiro, RJ, Brazil

[6]Laboratorio de Bioquímica de Resposta ao Estresse, Instituto de Bioquímica Médica, Universidade Federal do Rio de Janeiro, Rio de Janeiro, Brazil

[7]Laboratório de Microbiologia Celular, Instituto Oswaldo Cruz, Fundação Oswaldo Cruz, Rio de Janeiro, Brazil

[8]Departamento de Bioquímica, Escola Paulista de Medicina, Universidade Federal de São Paulo, São Paulo, SP, Brazil

[9]Departamento de Farmacociências, Universidade Federal de Ciências da Saúde de Porto Alegre, Porto Alegre, RS, Brazil

[10]Departamento de Parasitologia, Instituto de Ciências Biomédicas, Universidade de São Paulo, São Paulo, Brazil

[11]Instituto Nacional de Ciência e Tecnologia - Entomologia Molecular, Rio de Janeiro, RJ, Brazil

[12]Vector Biology Section, Laboratory of Malaria and Vector Research, National Institute of Allergy and Infectious Disease, Rockville, United States

* Corresponding Author: Itabajara da Silva Vaz Jr., Centro de Biotecnologia, Universidade Federal do Rio Grande do Sul, Porto Alegre, Brazil. E-mail: Itabajara.vaz@ufrgs.br

Table S1: Hyperlinked spreadsheet of transcripts from *Rhipicephalus microplus*. (Excel Spreadsheet)

Table S2: Functional classification of ovary-overexpressed transcripts (>5 x compared to other tissues) from *Rhipicephalus microplus*

Class	CDS	RPKM	CDS (%)	RPKM (%)
Secreted	171	14112	15.7	17.4
Nuclear regulation	31	13899	2.8	17.2
Unknown	218	11113	20.0	13.7
Unknown, conserved	93	6141	8.5	7.6
Signal transduction	107	4470	9.8	5.5
Metabolism, lipid	20	3658	1.8	4.5
Protein modification machinery	25	3273	2.3	4.0
Extracellular matrix/cell adhesion	19	3029	1.7	3.7
Oxidant metabolism/detoxification	15	2747	1.4	3.4
Secreted, mucins	8	2532	0.7	3.1
Transporters/storage	39	2395	3.6	3.0
Proteasome machinery	39	2203	3.6	2.7
Transcription machinery	96	2100	8.8	2.6
Pathogen origin	1	1742	0.1	2.2
Vertebrate origin	23	1653	2.1	2.0
Secreted proteinase inhibitor	10	1617	0.9	2.0
Protein synthesis machinery	16	680	1.5	0.8
Transposable element	46	577	4.2	0.7
Cytoskeletal	20	447	1.8	0.6
Metabolism, carbohydrate	13	432	1.2	0.5
Secreted protease	5	405	0.5	0.5
Transcription factor	16	395	1.5	0.5
Storage	2	249	0.2	0.3
Immunity	8	238	0.7	0.3
Signal transduction, apoptosis	3	213	0.3	0.3
Protein export machinery	15	201	1.4	0.2
Metabolism, nucleotide	9	160	0.8	0.2
Metabolism, amino acid	6	135	0.6	0.2
Metabolism, energy	5	81	0.5	0.1
Viral	7	49	0.6	0.1
Metabolism, intermediate	3	43	0.3	0.1

Table S3: Functional classification of embryo-overexpressed transcripts (>5 x compared to other tissues) from *Rhipicephalus microplus*

Class	CDS	RPKM	CDS (%)	RPKM (%)
Transposable element	28	346461	8.1	91.6
Unknown	89	8635	25.6	2.3
Secreted	73	7870	21.0	2.1
Unknown, conserved	21	7277	6.1	1.9
Vertebrate origin	18	2748	5.2	0.7
Extracellular matrix/cell adhesion	12	1308	3.5	0.3
Secreted, lipocalins	20	1207	5.8	0.3
Protein modification machinery	4	972	1.2	0.3
Signal transduction	13	571	3.7	0.2
Proteasome machinery	35	455	10.1	0.1
Transporters/storage	2	205	0.6	0.1
Protein export machinery	5	192	1.4	0.1
Metabolism, lipid	3	119	0.9	0.0
Bacterial	2	111	0.6	0.0
Transcription machinery	8	90	2.3	0.0
Oxidant metabolism/detoxification	4	48	1.2	0.0
Protein synthesis machinery	6	26	1.7	0.0

Table S4: Functional classification of salivary gland-overexpressed transcripts (>5 x compared to other tissues) from *Rhipicephalus microplus*.

Class	CDS	RPKM	CDS (%)	RPKM (%)
Secreted proteinase inhibitor	14	75078	4.4	31.2
Secreted	99	61030	31.0	25.3
Secreted, lipocalins	39	42412	12.2	17.6
Unknown, conserved	13	21244	4.1	8.8
Unknown	54	15920	16.9	6.6
Signal transduction	8	6857	2.5	2.8
Transporters/storage	11	4956	3.4	2.1
Transcription machinery	2	4204	0.6	1.7
Protein modification machinery	22	3539	6.9	1.5
Metabolism, carbohydrate	5	1505	1.6	0.6
Oxidant metabolism/detoxification	5	1380	1.6	0.6
Metabolism, nucleotide	3	1107	0.9	0.5
Transposable element	11	497	3.4	0.2
Vertebrate origin	4	395	1.3	0.2
Extracellular matrix/cell adhesion	8	392	2.5	0.2
Secreted protease	6	294	1.9	0.1
Proteasome machinery	4	69	1.3	0.0
Metabolism, amino acid	1	55	0.3	0.0
Cytoskeletal	2	20	0.6	0.0
Bacterial	3	10	0.9	0.0

Table S5: Functional classification of fat body-overexpressed transcripts (>5 x compared to other tissues) from *Rhipicephalus microplus*

Class	CDS	RPKM	CDS (%)	RPKM (%)
Secreted	55	70940	35.7	61.3
Unknown	15	12208	9.7	10.6
Viral	1	10781	0.6	9.3
Signal transduction	4	5373	2.6	4.6
Extracellular matrix/cell adhesion	14	4150	9.1	3.6
Secreted, lipocalins	9	2448	5.8	2.1
Unknown, conserved	7	1978	4.5	1.7
Secreted proteinase inhibitor	2	1769	1.3	1.5
Secreted, mucins	1	1336	0.6	1.2
Immunity	9	1009	5.8	0.9
Transcription machinery	3	748	1.9	0.6
Storage	1	600	0.6	0.5
Protein modification machinery	3	523	1.9	0.5
Metabolism, lipid	11	479	7.1	0.4
Oxidant metabolism/detoxification	8	423	5.2	0.4
Metabolism, amino acid	4	392	2.6	0.3

Table S6: Functional classification of digestive cells-overexpressed transcripts (>5 x compared to other tissues) from *Rhipicephalus microplus*

Class	CDS	RPKM	CDS (%)	RPKM (%)
Secreted	127	812178	20.0	61.2
Unknown	93	152133	14.7	11.5
Unknown, conserved	28	84352	4.4	6.4
Immunity	24	75751	3.8	5.7
Oxidant metabolism/detoxification	67	54072	10.6	4.1
Secreted proteinase inhibitor	21	53392	3.3	4.0
Secreted protease	28	27536	4.4	2.1
Protein modification machinery	27	26852	4.3	2.0
Metabolism, lipid	30	8978	4.7	0.7
Secreted, lipocalins	14	5467	2.2	0.4
Signal transduction	45	4762	7.1	0.4
Secreted, mucins	3	3379	0.5	0.3
Extracellular matrix/cell adhesion	19	3039	3.0	0.2
Metabolism, nucleotide	4	2701	0.6	0.2
Signal transduction, apoptosis	4	2202	0.6	0.2
Transporters/storage	27	1707	4.3	0.1
Vertebrate origin	15	1704	2.4	0.1
Transcription machinery	2	1144	0.3	0.1
Proteasome machinery	4	843	0.6	0.1
Storage	4	780	0.6	0.1
Metabolism, carbohydrate	15	707	2.4	0.1
Transposable element	11	656	1.7	0.0
Protein export machinery	8	608	1.3	0.0
Metabolism, energy	4	453	0.6	0.0
Metabolism, amino acid	6	388	0.9	0.0
Metabolism, intermediate	1	239	0.2	0.0
Cytoskeletal	2	102	0.3	0.0

Table S7: Functional classification of transcripts 5x more expressed in partially engorged than in fully engorged digestive cells from *Rhipicephalus microplus*.

Class	CDS	RPKM	CDS (%)	RPKM (%)
Secreted	48	318719	31.2	78.5
Unknown	26	49137	16.9	12.1
Unknown, conserved	7	12848	4.5	3.2
Immunity	4	9647	2.6	2.4
Protein modification machinery	5	5912	3.2	1.5
Secreted protease	1	2962	0.6	0.7
Extracellular matrix/cell adhesion	4	1264	2.6	0.3
Oxidant metabolism/detoxification	13	948	8.4	0.2
Metabolism, nucleotide	1	863	0.6	0.2
Proteasome machinery	3	826	1.9	0.2
Transporters/storage	7	681	4.5	0.2
Secreted, lipocalins	8	650	5.2	0.2
Signal transduction	7	473	4.5	0.1
Transposable element	6	331	3.9	0.1
Metabolism, lipid	7	272	4.5	0.1
Storage	1	152	0.6	0.0
Metabolism, amino acid	2	75	1.3	0.0
Secreted, mucins	1	71	0.6	0.0
Secreted proteinase inhibitor	2	52	1.3	0.0
Protein export machinery	1	23	0.6	0.0

Table S8: Functional classification of transcripts 5x more expressed in fully engorged digestive cells than in partially engorged digestive cells from *Rhipicephalus microplus*.

Class	CDS	RPKM	CDS (%)	RPKM (%)
Secreted	17	138753	17	46.1
Oxidant metabolism/detoxification	8	43006	8	14.3
Secreted proteinase inhibitor	6	37865	6	12.6
Immunity	12	34290	12	11.4
Unknown	18	27944	18	9.3
Protein modification machinery	7	7256	7	2.4
Secreted protease	6	4287	6	1.4
Metabolism, lipid	5	3478	5	1.2
Signal transduction, apoptosis	3	2053	3	0.7
Vertebrate origin	7	975	7	0.3
Unknown, conserved	3	952	3	0.3
Transporters/storage	3	80	3	0.0
Signal transduction	2	48	2	0.0

Table S9**.** Sequences in immune system category overexpressed (fold-change ≥5) in fat body (FB), digestive cells (DIG), ovaries (OV), or salivary glands (SG) from *Rhipicephalus microplus*.

CDS ID	Annotation	Tissue overexpressed
Rm-26573/Rm-26574/Rm-52480	microplusin	FB
Rm-14039/Rm-14040/Rm-73312	α-macroglobulin	
Rm-77302/Rm-76915	ixoderin	
Rm-8119	defensin	DIG
Rm-25766/Rm-95726	TNF-α receptor	
Rm-79342/Rm-65368/Rm-65369	γ-interferon inducible	
Rm-65192	lysosomal thiol reductase	
Rm-40113	C-C motif chemokine 5	
Rm-7355/Rm-3360/Rm-7351/ Rm-7352/Rm-7348/Rm-7458/ Rm-32800/Rm-7353/Rm-7349	ixoderin	
Rm-50824	membrane glycoprotein lig-1	
Rm-19492/Rm-16273/Rm-136776/ Rm-7097/Rm-17234	ML domain protein	
Rm-42466	granulin	
Rm-27599	microplusin	OV
Rm-7822	α-macroglobulin	
Rm-48095/ Rm-48096	ixoderin	
Rm-51466/ Rm-5160/Rm-20167/ Rm-66225	TNF-α receptor	
Rm-164126	membrane glycoprotein lig-1	
Rm-80487	Spaetzle-like protein 4	
Rm-164727/Rm-164728	possible membrane-associated motif in LPS-induced TNF-α factor	
Rm-47239	immunoglobulin G-binding protein 1	
Rm-23502	members of chemokine-like factor super family	SG
Rm-149520	preprodefensin	

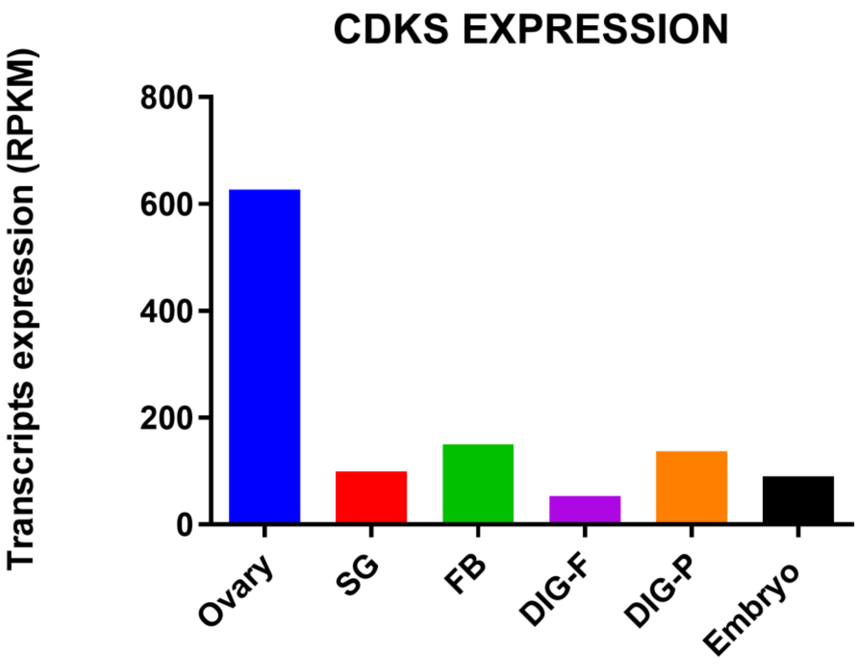

Figure S1: Expression analysis in the transcriptome of *Rhipicephalus. microplus.* The graph represents the total cyclin-dependent kinases (CDKs) expression by tissues from females and the CDKs expression in digestive cells from partially (DIG-P) and fully engorged females (DIG-F). Expression was measured by RPKM.

Video S1: Isolation of digestive cells of *Rhipicephalus microplus*. Digestive cells from partially engorged females (DIG-P) and fully engorged females (DIG-F) were isolated as previously described (Lara et al., 2005). Essentially, ticks were dissected in Petri dish containing sterile PBS, midguts were isolated, opened gently with tweezers and cells were detached from the gut wall manually with tweezers.
